# Hay Fever

**Published:** 1907-10

**Authors:** E. S. M Kee

**Affiliations:** Cincinnati


### ABSTRACTS.

### Hay Fever

By E. S. M KEE, M. D., Cincinnati.
(*Cincinnati Lancet Clinic*).

ABOUT the time that the hay is all carefully stored away it the mow out of the rain then we have to combat hay fever. The proper time to write on this subject would be after the close of the season, for hindsight is much better than foresight on this as well as many other questions. I think we made some distinct advance on this line last season, and to recall attention to the study of this trouble, to express the hope that we will make still greater advance this season, and possibly add my mite is the object of this paper.

### AIR FILTRATION.

The filtration of the air which enters the nose is a matter of much importance. This is why the growth of hair is there and is why many ointments help simply by stopping the pollen which the air contains. Bits of cotton have been inserted in the nose with some benefit. Apparati have been used consisting of rings made of cork, hard rubber, or, best, of German silver. These rings have stretched over them thin linen or chiffon silk. They are placed within the nostril perhaps a half-inch up, so that all the inspired air must pass through them. Many patients obtain much relief by sleeping with a thin moistened handkerchief over the face.

### HAY FEVER AMENABLE TO NO SPECIFIC TREATMENT.

H. Holbrook Curtis considers hay fever a disorder amenable to no treatment of a specific nature. About one-third of the cases of hyperesthetic rhinitis are from other causes and other pollens than the ragweed. About one-third of the cases supposed to be due to pollen reaction may be relieved by constitutional and surgical methods of treatment. Predisposition to attack in these cases being due to definite causes, would suggest the view that induced enervation of the sympathetic is an important factor in the etiology. Primary intoxications may take place from pollen toxines in cases where the sympathetic system is not previously enervated. These cases theoretically should react to antitoxine treatment. The consensus of opinion today is against pollantin, though the observers instructed personally by Prof. Dunbar endorse unqualifiedly the great benefit to be derived from the treatment. Medically, the suprarenal capsule products hold the first place in the treatment of hypertrophic rhinitis. The importance of constitutional treatment as an adjunct to any local application is of supreme importance. The best of all treatments yet found is the climatic, with primary attention to the nasal condition.

### THE USE OF MORPHINE AND COCAINE IN HAY ASTHMA.

Though most of the text-books call attention to the great danger from the administration of morphine and cocaine in asthma, this fear is probably overdrawn. Most practitioners find that it is necessary to use one or both in severe cases before relief is obtained, and it is generally found that when the distress disappears the desire for these insidious remedies is also gone. If carefully watched and kept under the control of the physician these drugs are not very dangerous in this disease and form a very necessary part of the treatment. Especially is this true of those cases where the attacks occur at rare intervals, that it is safe to use morphine and cocaine with caution. More care is necessary in those cases where the status asthmaticus is more or less constant. It is an important thing to know that quite small doses will furnish a remarkable amount of relief, and, in fact, less than the ordinary sized doses are required. When we note that the patient is demanding larger doses, then is the time to reduce, stop or switch the drug. To repeat: these two remedies are of much use in these conditions and are not as harmful to the patient as most of us believe in hay asthma.

### THYMOL IODIDE IN HAY FEVER.

This has been found of much service. It is used on the theory that the point of departure of the reflex irritation which produces hay fever is situated in the mucous membrane of the antrum of Highmore. The thymol iodide should be insufflated through the orifice of the maxillary sinus situated in the middle meatus of the nasal chambers. It should be applied with a fine powder insufflator with a curved cannula. The relief is quite marked and of long duration, though in some it is necessary to repeat the application.

### THE NERVOUS SYMPTOMS.

These are of great prominence in hay fever, and should be given due consideration. All hurry and worry should be avoided. The patient should live the simple life as to habits, diet and sleep. Rich foods are especially to be avoided. Dark glasses add to the tranquility of the eyes.

### DUNBAR'S POLLANTIN.

This remedy, though not proving as beneficial as its advocates thought it would, yet it is distinctly palliative in 30 to 60 per cent. In about 30 per cent. of cases both pollantin and suprarenalin fail to give any effect. The nose should be thoroughly cleansed with alkaline solutions. The application should be thoroughly made and the patient should lie down for ten or fifteen minutes after the application.

SUPRARENALIN.

This remedy is best given in tablets put up with a very small amount of milk sugar, each tablet containing from one-fortieth to one-tenth grain. This should be placed upon the tongue and allowed to melt there. It is not proper to swallow it as a pill. This can be repeated in the course of from ten minutes to two hours according to necessity. The need of repeating depends on whether the patient remains at home or goes out in the dust, bright sun, or in the country. It varies also in different individuals.

The following are some good formulæ:

| | |
|---|---|
| Suprarenalin, | gr. 1-6 or 0.01 |
| Adeps lannæ | |
| Adepis benzoati, aa, | dr. i or 4.00 |
| Petrolati, | |

Mix well and dispense in tubes.

| | |
|---|---|
| Suprarenalin, | gr. 1-6 or 0.01 |
| Adepis lannæ, | dr. i or 4.00 |
| Unguenti zinci oxidi, | drs. ii or 8.00 |
| Ol. olivæ, | drs. iiss or 10.00 |

M. S.—Dispense in tubes and apply in the nasal passages.

| | |
|---|---|
| Suprarenalin, | 0.01 |
| Zinci stereat. comp., | 1.00 |
| Magnesiæ carbonatis, | 9.00 |

M. S.—Triturate well and use as a snuff.

| | |
|---|---|
| Suprarenalin, | 0.01 |
| Bismuth subnitratis, | 3.00 |
| Zinci oxidii, | 3.00 |
| Zinci stearatis, | 2.00 |

M. Triturate well and use as a snuff or in a powder blower.

A good spray formula:

| | |
|---|---|
| Cocaine hydrochl., | 1.00 |
| Adrenalin (1:1,000), | 4.00 |
| Normal salt sol., | 60.00 |

M. S.—Use as a spray in the nose.

| | |
|---|---|
| ℞-Cocaine hydrochloratis | 1.00 |
| Sol. Adrenaline | (1-1,000) 10.00 |
| Sodii Chloridii | 0.75 |
| Aquæ Destillatæ ad | 100.00 |

M. S.—Use as spray in the nose reclining for 15 minutes thereafter.

For the asthmatic paroxysm—in hay fever: give 1|10 grain tablet, on the tongue, of suprarenalin, allow it to dissolve and repeat in 10 or 20 minutes if necessary. Suprarenalin or adrenalin solution 1:1,000 or diluted five or ten times may be applied to the nose by spray or on cotton, the patient taking the recumbent position for 10 or 15 minutes to allow the medicine to gravitate to all parts of the nose. The quickest means of obtaining relief is to administer suprarenalin or adrenalin hypodermically in ten or twenty minim doses. Most practitioners find it necessary to use morphine or cocaine or both in severe cases before relief is obtained. It is generally found that when the distress is gone the desire for these insidious remedies disappears. These two remedies are of much use in these conditions and are not as dangerous in inducing a habit in hay asthma as most of us would believe.

ATROPHINE SULPHATE AND HYOSCINE HYDROBROMATE.

These remedies in very small doses are beneficial—for instance, given in doses of from 1-2500 to 1-500 grain.

DURING THE ASTHMATIC PAROXYSM

give 1-10 grain tablet suprarenalin on the tongue and repeat in ten or twenty minutes if necessary. Suprarenalin or adrenalin solution 1.:1,000 may be applied to the nose by spray or on cotton. The quickest means of obtaining relief is to administer suprarenalin or adrenalin solution, 1:1,000, hypodermically, in ten to twenty-minim doses.

REPETITION OF PRESCRIPTIONS.

Any contract between physician and druggist whereby the former is to receive a commission from the latter, for sending prescriptions to him, is illegal and against public policy.

MISCELLANEOUS.

The first essential in the successful cure of sciatica, the hip gout of Pliny, is a thorough knowledge of the individual patient in hand. An exact diagnosis of the conditions is one of the first and last means of cure.

As treatment in internal cephalhematoma has not yet been attempted; as the internal is almost always associated with the external variety, the latter situated directly over the former, in the writer's opinion it would be advisable to trephine the skull to evacuate the tumor.—E. S. McKee, M.D., Cincinnati *Lancet-Clinic,* vol ii, 1883, p 322; also first and second editions of Wood's Reference Handbook of the Medical Sciences. This operation is now being recommended as an original idea.

There is an evident connection between the nose and the sexual organs which is especially well marked in dogs. Sexual excesses are a causative factor in rhinitis as are also smoking and alcoholic excesses. In all cases of recurrent coryza where cocaine is prescribed do not forget to ad "Ne repetature."

